# Development of Core KASP Marker Panel for DNA Fingerprinting of Pear (*Pyrus* spp.) Germplasm Collections

**DOI:** 10.3390/plants15203111

**Published:** 2026-10-11

**Authors:** Dan Qi, Luming Tian, Hongliang Huo, Jiayu Xu, Simeng Zhang, Ying Zhang, Chao Liu, Xingguang Dong, Yufen Cao

**Affiliations:** 1 Research Institute of Pomology, Chinese Academy of Agricultural Sciences, Xingcheng 125100, China; 2 Key Laboratory of Germplasm Resources Utilization of Horticultural Crops, Ministry of Agriculture and Rural Affairs, Xingcheng 125100, China

**Keywords:** *Pyrus*, KASP, genotype, DNA fingerprint, genetic characteristics

## Abstract

Accurate varietal identification remains a persistent issue in pear germplasm conservation, creating obstacles in breeding parent selection and impeding progress in elite cultivar research and cultivation. This study aims to develop a set of highly polymorphic single-nucleotide polymorphism (SNP) markers for pear variety identification. Based on the design criteria for highly polymorphic KASP primers, 34 primer pairs were synthesized, of which 25 produced reliable genotyping results, showing clear separation among different genotypes with minimal missing data. The 269 pear accessions were successfully genotyped, with observed heterozygosity (Ho) ranging from 0.33 to 0.56, polymorphism information content (PIC) values between 0.336 and 0.375, and minor allele frequency (MAF) ranging from 0.31 to 0.50. A total of 257 distinct genotypes were obtained from 269 pear accessions, among which two groups of known bud mutation were consistent, and ten groups of potential synonym accessions were detected. The cluster analysis based on the 25 KASP markers separated the 269 pear accessions into two clusters, which showed a broad association with taxonomic background and origin. This study shows that this CoreSNP-KASP marker set can be used for preliminary molecular screening of pear accessions and provides useful information to support subsequent breeding programs.

## 1. Introduction

Pear originated in China, which served as the differentiation center of Oriental pear and one of the major diversity centers for cultivated pear, and is characterized by abundant genetic resources and a long history of cultivation. Pear plants exhibit a high level of self-incompatibility, lack reproductive isolation among species, frequently undergo interspecific hybridization, and possess a complex genetic background. Currently, at least 1000 pear germplasm accessions are preserved as trees in the National Germplasm Repository of Pear and Apple (Xingcheng, China), which are propagated through clonal cuttings and are not suitable for storage as seeds. Pear varieties are frequently exchanged between different regions, and some introduced or local varieties lack exact scientific names, which inevitably leads to the phenomenon of synonyms or homonyms. Therefore, developing a cost-effective, efficient, and accurate method for variety identification is of great significance for improving the management efficiency and variety protection capabilities for pear germplasm resources.

Various DNA marker technologies have been developed to study the genetic diversity, population structure, and fingerprints of pear varieties [1,2,3,4]. Among them, the International Union for the Protection of New Varieties of Plants (UPOV) recommends SSR and SNP markers as the preferred markers for crop variety identification [5]. Over the past two decades, SSR markers have been extensively utilized for the identification of pear cultivars and the characterization of pear germplasm [6,7,8,9]. Nevertheless, SSR genotyping is hampered by properly identifying precise information in terms of multiple alleles per locus.

The reduced sequencing cost and increased throughput of next-generation sequencing technology have promoted the large-scale discovery of genome-wide SNP markers. Compared with SSR markers, SNPs are more abundant and evenly distributed across the genome, making them ideal for high-throughput genotyping and genome-wide association studies. In pear, current SNP genotyping approaches mainly include high-density arrays [10,11] and Multiple Nucleotide Polymorphism (MNP) markers [12]. Each platform has its own limitations: genotyping arrays are expensive and instrument-dependent, while MNP marker development and experimental workflows are relatively complicated. To date, low-density SNP assays using PCR-based detection for pear with high accuracy and low cost and only requiring routine equipment in most labs are still lacking.

KASP offers cost-effective and scalable flexibility in applications that require small-to-moderate numbers of markers [13]. Currently, KASP assays are employed in many research fields, such as fine QTL mapping for MAS [14,15,16] and quality control genotyping [17,18]. KASP markers have also been employed in fingerprint construction for crop species, including coffee [19], grape [5], wheat [20], bottle gourd [21], cauliflower [22], cowpea [23], and radish [24]. This SNP genotyping platform can therefore be used for genotyping pear germplasm and establishing DNA fingerprints of major cultivars.

This study aims to develop SNP markers using whole-genome resequencing data from 495 pear accessions and to convert highly polymorphic SNP loci into KASP markers. We will further screen a minimal panel of informative CoreSNP markers and construct a DNA fingerprint database for 269 pear germplasm accessions maintained in the repository based on KASP genotyping data. The outputs will facilitate the identification and utilization of pear germplasm, enable full exploitation of China’s abundant pear germplasm resources, and lay a material basis for pear variety improvement and breeding.

## 2. Results

### 2.1. SNP Screening and Identification of High-Quality KASP Markers

The resequencing dataset comprising 495 pear samples contained 11,031,864 biallelic SNP loci, with an average sequencing depth of 10.72× [25]. Further filtering and genotyping of this dataset were carried out in accordance with KASP primer design principles. A total of 453,801 SNP loci met the criteria of having no other mutations within 100 bp upstream or downstream of the polymorphic site; of these, 34,282 SNP loci had an MAF > 0.4, and 27,274 SNP loci had an average sequencing depth of 10×. There were 11,015 SNPs with a sample missing rate of 0.

Using a Perl script [26], optimal loci combination tests were conducted on loci across 17 chromosomes, yielding one set of loci per chromosome, for a total of 333 loci. Of these, 140 were SNP loci where the sequences within 100 bp upstream and downstream of the variant site exhibited single-copy characteristics. Finally, optimal loci combination testing was conducted on the 140 loci, the selected combinations capable of distinguishing 495 samples in the dataset. Considering potential genotyping failure in practical KASP assays, three alternative loci combinations were designed. After excluding redundant loci shared among combinations, these three panels represented a total of 34 unique loci. Detailed information for each locus in the three combinations, including chromosomal positions reflecting their genomic distribution, is listed in Supplementary Table S1.

**Figure 1:**
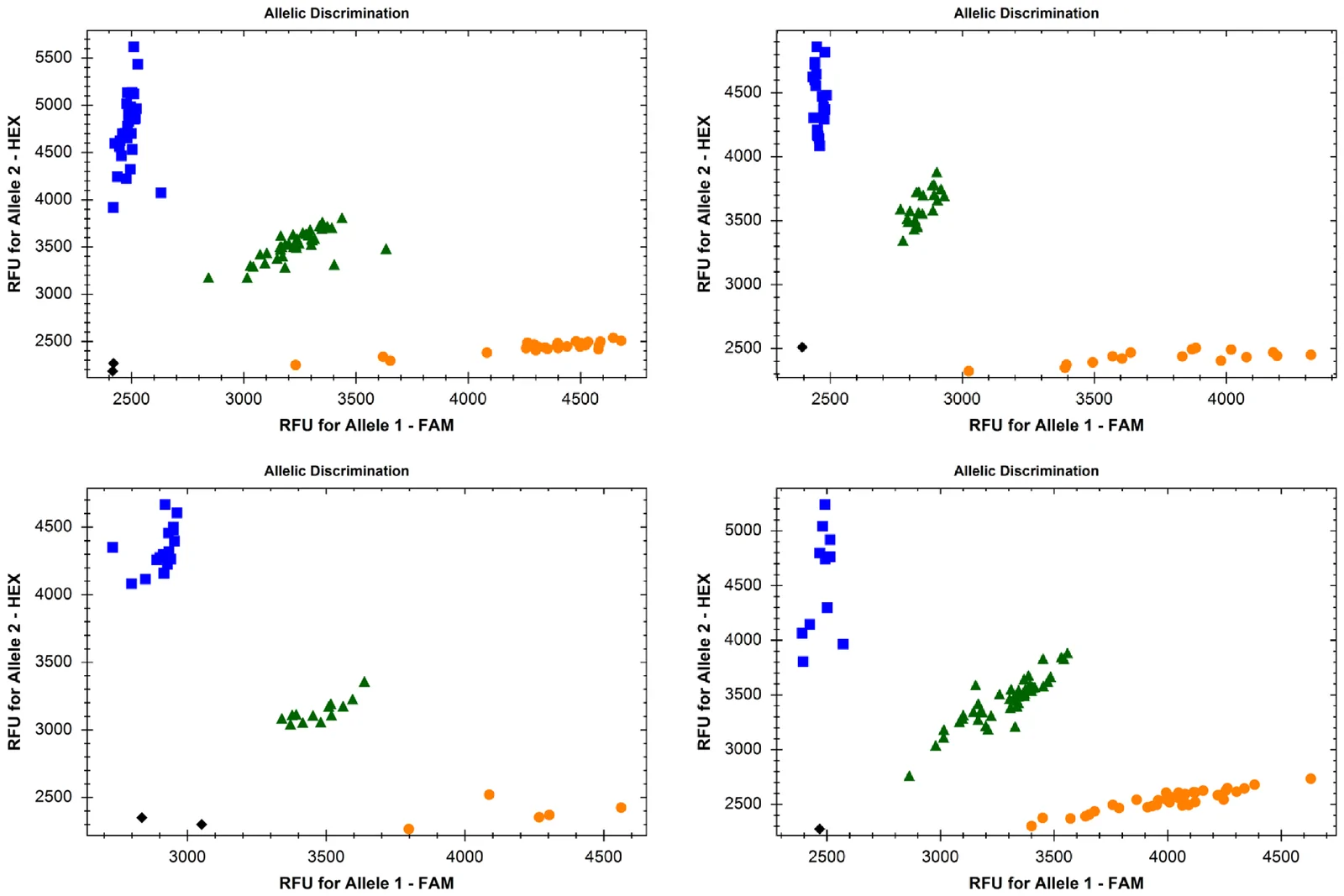
Partial sample genotype plots of 4 representative KASP markers. Blue squares and yellow dots denote homozygous alleles, green triangles denote heterozygous alleles, and black rhombi represent the negative control (H_2_O).

The PCR amplification results showed that 25 primer pairs yielded genotyping data capable of reliably distinguishing three genotypes: two homozygous genotypes (AA and BB) and one heterozygous genotype (AB), with well-separated genotype clusters and low missing rates, while nine primer pairs (see Table S1) failed to accurately call the genotypes of samples. The 25 primer sequences are shown in Supplementary Table S2. The dot plot shows that each pair of primers detected three genotypes, including two homozygous genotypes (AA and BB) and one heterozygous genotype (AB) (Figure 1). The boundaries between different genotypes were clearly delineated, and the occurrence of missing values was minimal.

**Figure 2:**
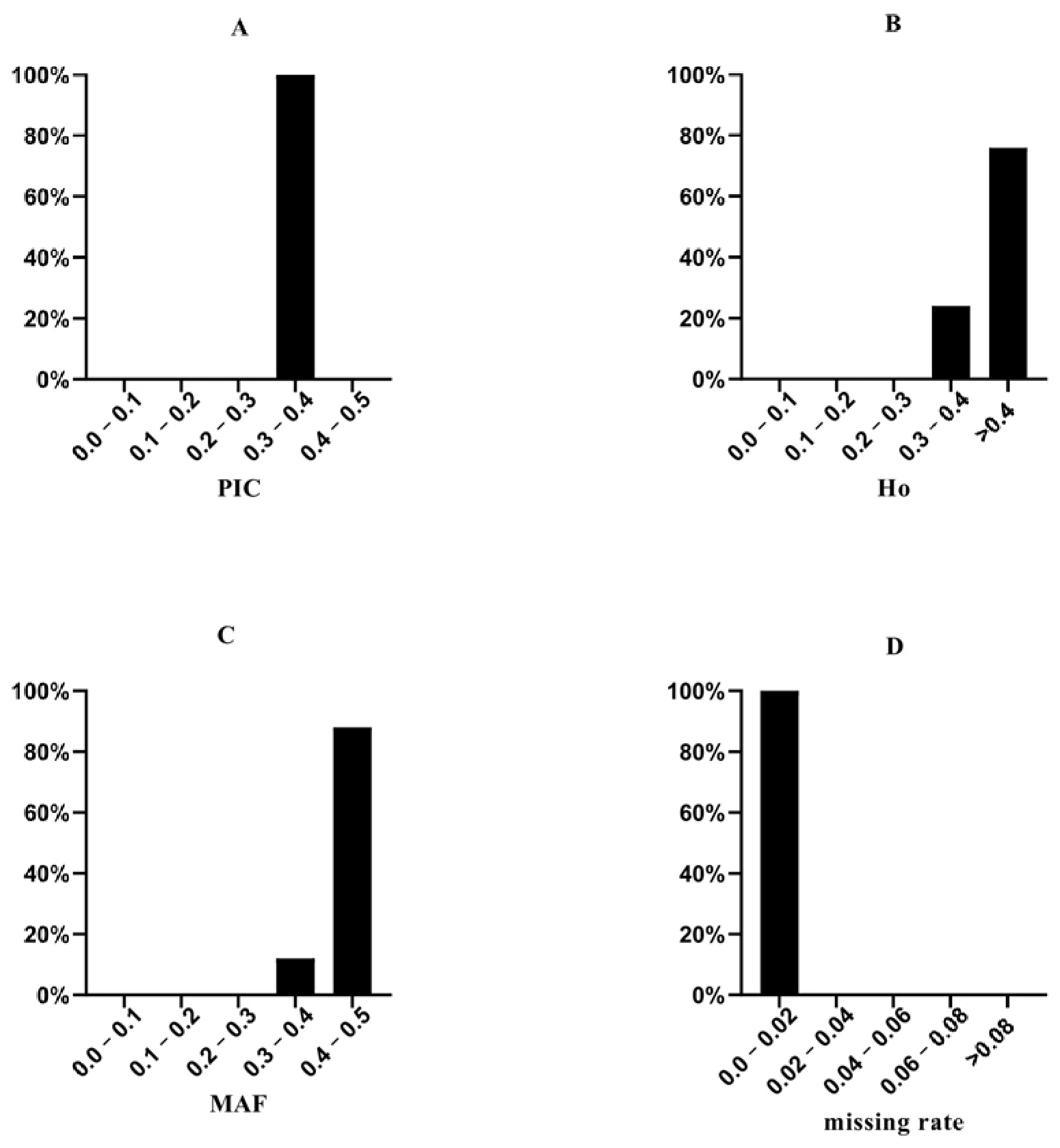
PIC (**A**), Ho (**B**), MAF (**C**), and missing rate (**D**) values for the 25 KASP markers.

Because SNP loci are biallelic, their PIC values are limited to the range of 0 to 0.5. For the 25 KASP markers analyzed, the PIC values ranged from 0.336 to 0.375 (Figure 2A). The observed heterozygosity (Ho) varied between 0.33 and 0.56, with 76% of markers showing Ho values above 0.4 (Figure 2B). The minor allele frequency (MAF) ranged from 0.31 to 0.5, and 88% of markers displayed MAF values greater than 0.4 (Figure 2C). The missing rate for the 25 primers ranged from 0 to 0.0074; all markers had a missing rate below 0.02 (Figure 2D) (Table 1).

### 2.2. Comparison of KASP Genotyping with Resequencing Results

**Figure 3:**
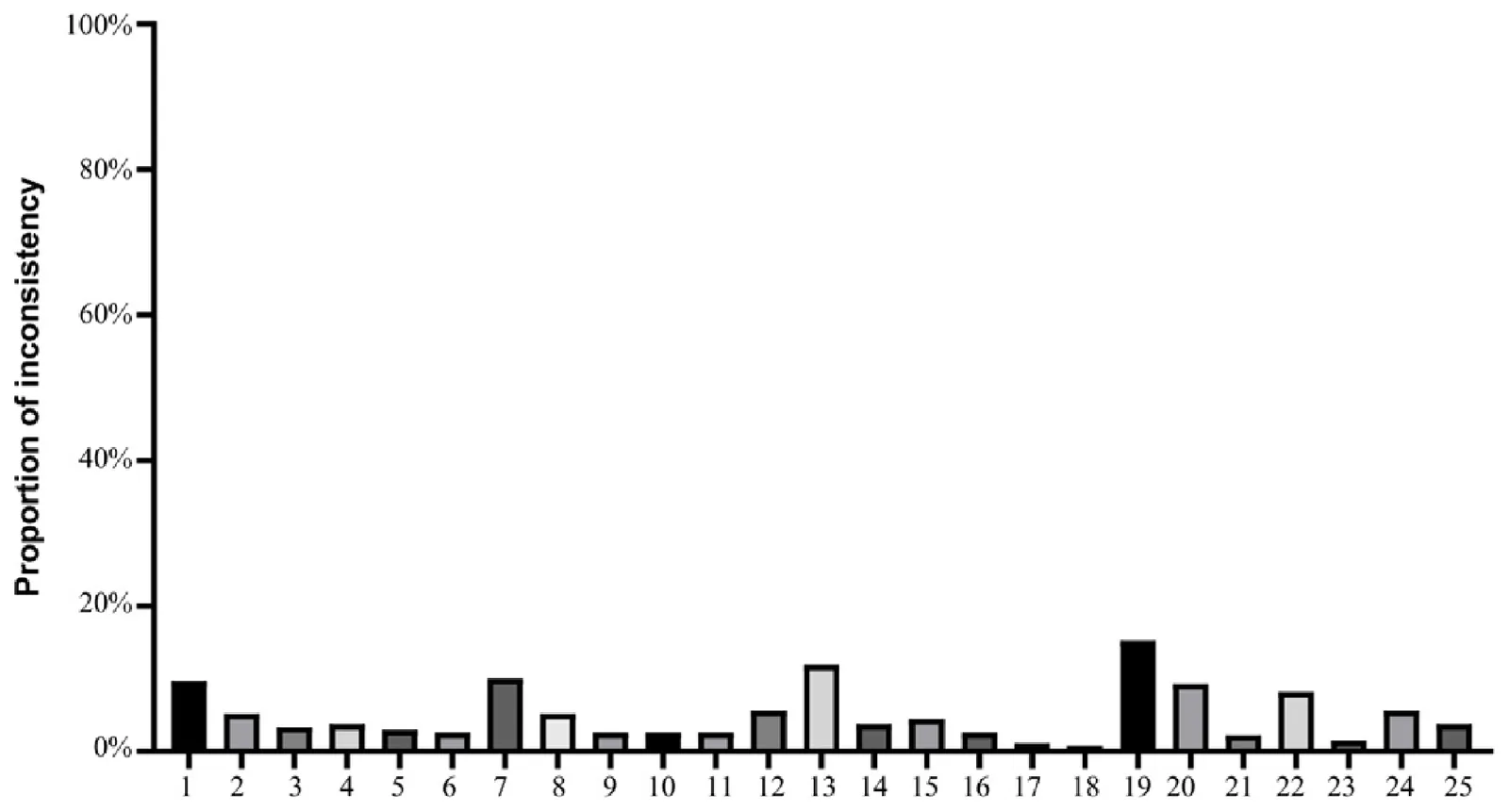
Genotyping inconsistency rate between 25 KASP markers and resequencing results for 269 pear germplasm accessions.

The genotyping results using the 25 KASP primers from 269 pear accessions were compared to the resequencing results, and the resequencing genotype results are provided in Table S3. A total of 340 genotype calls were found to be discordant with the resequencing results, accounting for 5.06% of all genotype calls, while the concordance rate reached 94.94%. Two replicate experiments were performed for the loci with inconsistent genotyping results, and the results remained consistent. The proportion of inconsistency for the 25 primers ranged from 0.74% to 15.24% (Figure 3). Three markers (Chr12_17378039, Chr9_508439, and Chr5_3714662) exhibited a significantly lower degree of agreement, with disagreement rates of 15.24%, 11.90%, and 10.04%, respectively. At the sample level, the KASP genotyping results of 81 samples were found to be completely consistent with the resequencing results, accounting for 30.11% of all tested accessions, and the number of samples with one to five locus inconsistencies was 90, 63, 20, 11, and 4, respectively. Among all discordant genotype calls, 212 corresponded to heterozygous loci and 128 to homozygous loci.

### 2.3. Population Analysis Using KASP Markers

**Figure 4:**
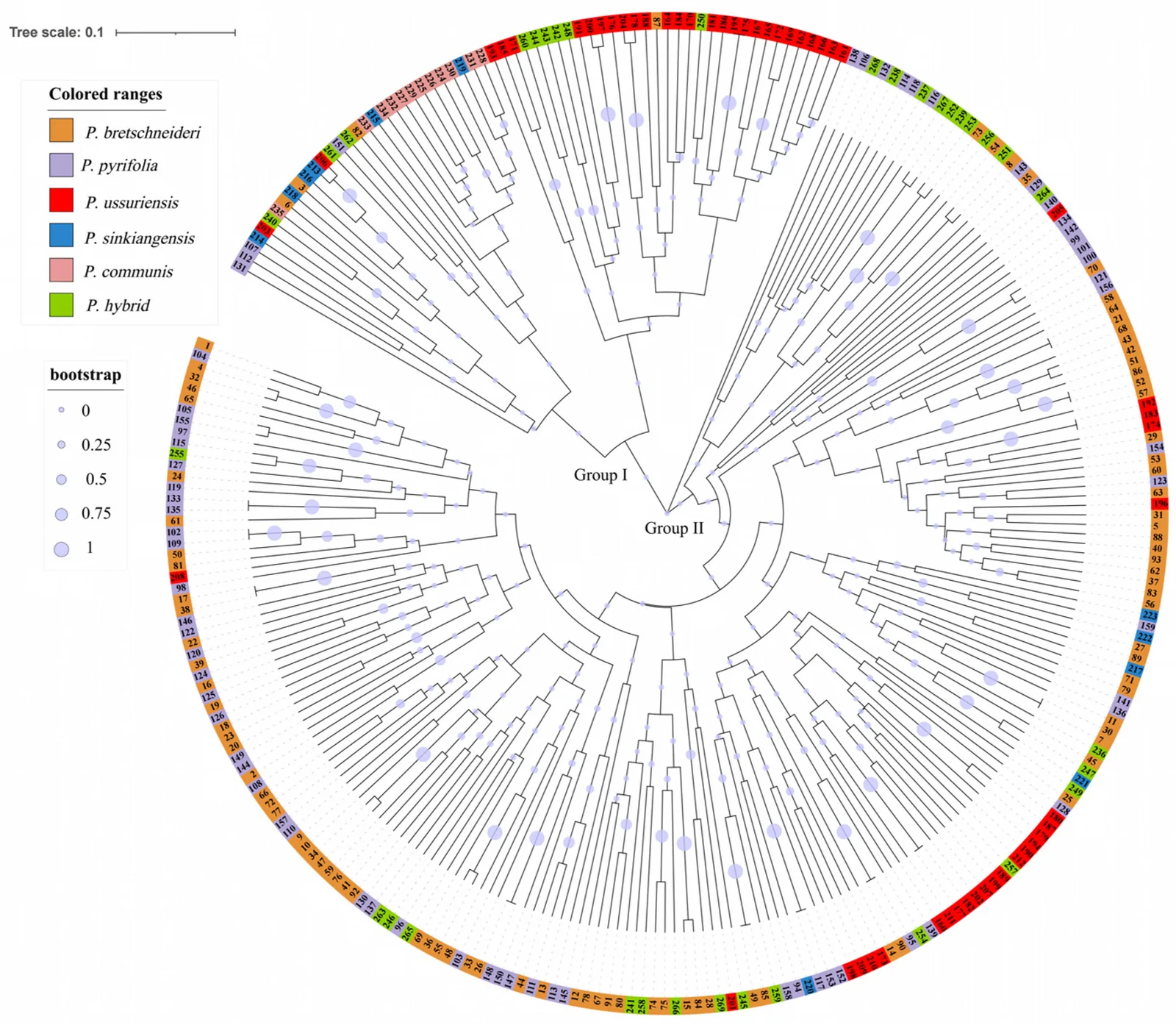
UPGMA tree of 269 accessions evaluated with the 25 core KASP markers.

To further evaluate the discriminatory power and genetic analytical capability of the core KASP markers, we constructed an UPGMA dendrogram of 269 pear germplasm resources based on the genotyping results using the 25 KASP markers (Figure 4). Cluster analysis separated the accessions into two groups, and multiple sub-groups existed inside each major cluster. Group I consisted of 63 accessions dominated by *P. communis* and Northeast China native *P. ussuriensis*, which produce soft and fragrant ripe fruits. This group also harbored *P. sinkiangensis* (a hybrid of Asian and European pears) and hybrid progeny of *P. ussuriensis*. Group II consisted of 206 accessions native to Asia, covering the germplasm of *P. bretschneideri*, *P. pyrifolia*, *P. ussuriensis* and *P. sinkiangensis*. This group included large-fruited crisp-fleshed *P. pyrifolia* and *P. bretschneideri* from the North China Plain and West Sichuan Plateau, as well as *P. ussuriensis* resources from Northwest China and northern Hebei, alongside *P. sinkiangensis* and *P. bretschneideri* germplasm distributed in the Yanshan Mountain range. Collectively, the genetic clustering of pear germplasm accessions shows a broad association with taxonomic background and origin.

**Figure 5:**
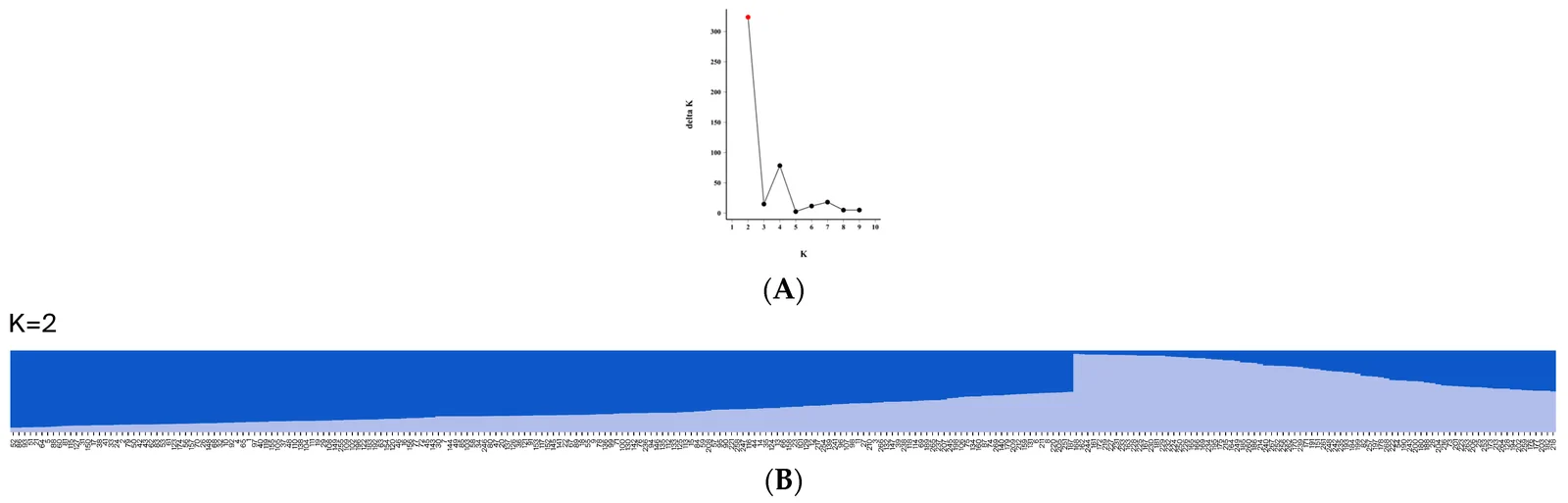
(**A**) Delta K (ΔK) values for 25 core KASP markers; (**B**) population structure analysis when K = 2.

We analyzed the population structure of 269 pear germplasm resources using genotyping data from the 25 KASP markers. The number of populations was determined according to the Delta K (∆K) method described by Evanno et al. [27]. When K = 2, ∆K reached its maximum value, indicating that the materials could be divided into two populations (Figure 5A,B). Accessions with Q ≥ 0.6 were assigned to Pop1 or Pop2; accessions with Q < 0.6 were regarded as admixed individuals. The Q values for 269 accessions are provided in Table S4. In general, 165 pear germplasm resources (61.34%) were assigned to pop1, and 62 pear germplasm resources (23.05%) were assigned to pop2. This grouping pattern was consistent with the two major branches observed in the UPGMA dendrogram, and the membership of each genetic group largely agreed with the clustering results. Collectively, these two complementary analyses corroborate the inferred genetic grouping of pear accessions.

### 2.4. Genotype Identification

A total of 257 distinct genotypes were detected in 269 samples using 25 pairs of primers with good genotyping results. The UPGMA dendrogram indicated that 12 groups were indistinguishable using these core KASP markers (Figure 4). Ten groups of accessions with identical genotypes were identified, and each group consisted of two germplasm accessions: ‘Zhengzhou E’li’ and ‘Yinbai’, ‘Hongtangli’ and ‘Hongtangli 2’, ‘Shuixiangli’ and ‘Xiali’, ‘Mili’ and ‘Jidanguan’, ‘Shimen Shuidonggua’ and ‘Fojianxi’, ‘Fengxian Jitui’ and ‘Puchengxue’, ‘Hanyuanbai’ and ‘Jinbabai’, ‘Banjinsu’ and ‘Kuixing Make’, ‘Sajin’ and ‘Shengxian Qiubai’, as well as ‘Fuyuanhuang’ and ‘Huangpi Shuiyabian’. These pairs of germplasm resources were suspected to be synonyms. Furthermore, genotyping analysis revealed identical genetic profiles between the two sets of known bud sports, ‘Qiuzi’ and ‘Tianqiuzi’, as well as between ‘Nanguo’ and ‘Dananguo’.

**Figure 6:**
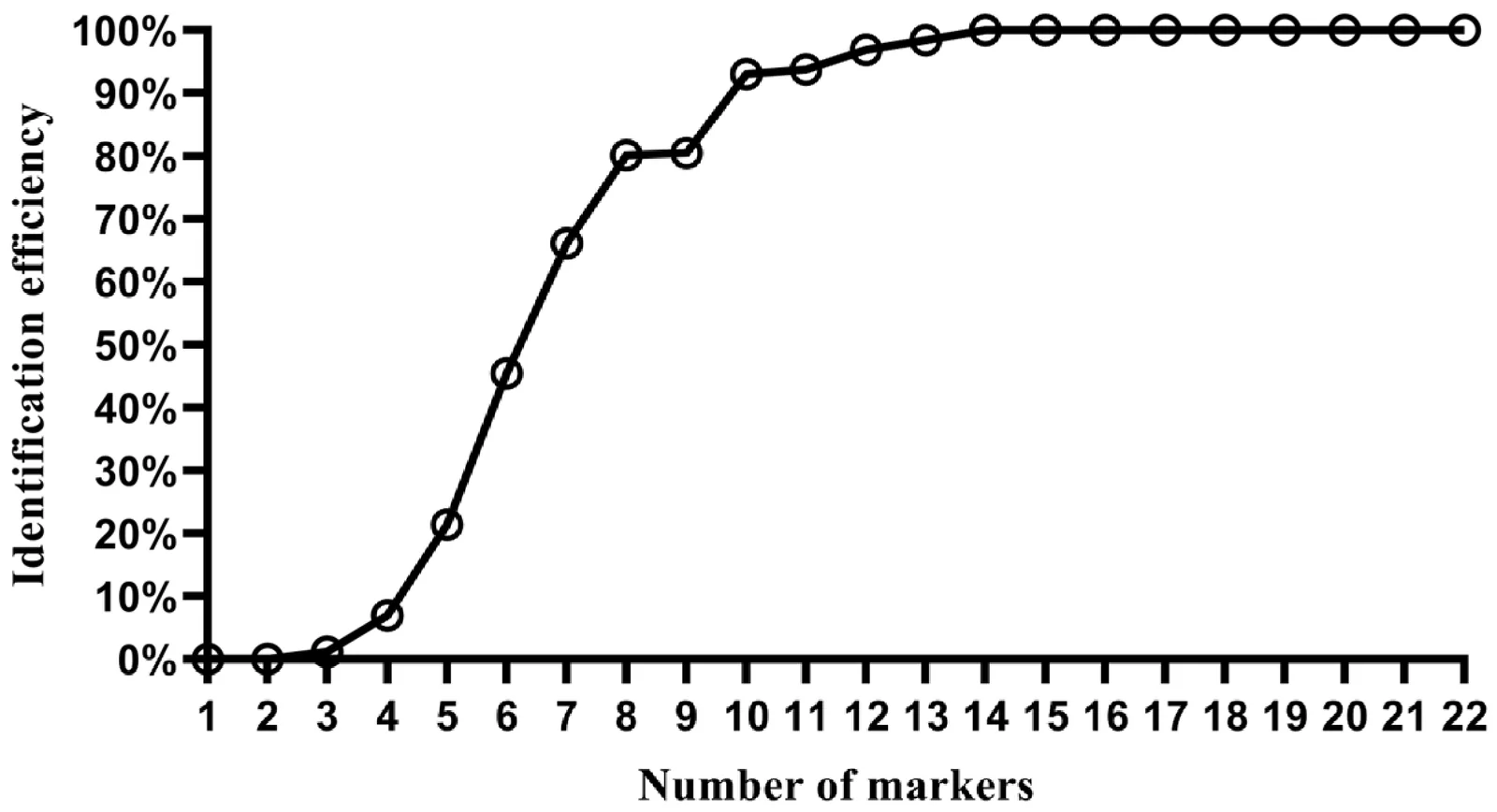
Cumulative KASP marker efficiency for the 257 pear germplasms.

The three markers Chr12_17378039, Chr9_508439 and Chr5_3714662, with high genotyping discordance between KASP and resequencing were excluded, leaving a total of 22 markers. The 22 KASP markers were sorted in descending order based on observed heterozygosity (Ho). The columns of the genotype matrix were reordered following this Ho ranking, and only unique genotypes were retained. Markers were then added sequentially according to the Ho order, and the germplasm discrimination power was recorded at each step. The top 14 markers (Supplementary Table S1) with the highest Ho values were sufficient to distinguish these 257 genotypes (Figure 6). This sequential selection identifies the first Ho-ranked marker set achieving full discrimination, without exhaustive testing of all alternative marker combinations.

### 2.5. Independent Validation Using 76 Pear Accessions

To evaluate the performance of this marker panel on materials not involved in marker screening, an independent panel consisting of 76 pear accessions was genotyped using the same 22 KASP assays. Initial identification with the top 14 markers failed to separate nine accessions assigned to four groups (2 + 2 + 2 + 3). Incorporating the remaining markers produced no change in the discrimination outcome. Genotyping results of the 76 accessions using the 22 KASP marker pairs are provided in Table S5.

### 2.6. SNP Fingerprint Construction

Analysis of 269 pear accessions identified 257 distinct genotypes, attributable to bud sports and putative synonymous accessions. Among these, 245 varieties exhibited unique genotypic characteristics. For instance, the genotyping result of ‘Liuyuexue’ was CTCCAGCCAGCCCTTTGTGATACTTTTCCTAACCCCAGAAAATG. The fingerprints of these 269 pear accessions based on the 22 markers are illustrated in Table S6.

## 3. Discussion

### 3.1. Efficiency of KASP Primers for Pear Accession Identification

In the present study, SNPs were screened according to the criteria proposed by Winfield et al. [26]. The optimal combination of core SNP loci enabled the complete discrimination of 495 pear germplasm accessions, with each accession exhibiting a unique genotypic profile. The strategy of employing a small set of core SNPs for variety identification has been widely applied in horticultural crops. For example, 40 KASP-SNP combinations were used to differentiate 96 tomato accessions [28], and 22 core SNPs could effectively distinguish 206 bottle gourd varieties [21]. These studies adopted similar strategies to achieve high-resolution variety identification with a limited number of markers, which helps reduce the cost of germplasm authentication.

In this study, three sets containing 34 primer pairs were designed and synthesized, among which 25 loci were finally selected due to their high polymorphism and clear distinction between homozygous and heterozygous genotypes, resulting in a conversion rate of 73.53%. As previously demonstrated, extensive homeologous and paralogous genomic sequences hinder the development of robust KASP markers in polyploid species [29]. Given that our pear germplasm contains triploid individuals, this constraint is also applicable to the present study. Previous studies reported different KASP marker conversion rates in other crops. A relatively high conversion rate of 85% was documented in cauliflower [22], and an even higher rate of 88.4% was found in Chinese cabbage [30]. In contrast, *Rubus* [31] presented a much lower conversion rate of only 24%. Encouragingly, we still achieved a moderate-to-high conversion rate despite these difficulties associated with polyploidy.

Furthermore, the 25 pairs of KASP markers were utilized for the identification of genotypes of 269 pear accessions. In comparison with the resequencing results, the concordance rate for 25 pairs of primers with 269 pear accessions was 94.94%; a similar value (95.8%) was obtained in apple [26]. Cao et al. [32] genotyped 105 germplasm accessions with 35 SNPs using the Sequenom MassARRAY platform. When validating their genotyping data against resequencing reads, they reported genotyping accuracies of 93.59% and 83.82% for homozygous and heterozygous SNP loci, respectively. Zhang et al. [33] randomly selected 3942 SNP loci identified by pear genome resequencing for verification via Sanger sequencing, and the results revealed that the accuracy of these SNPs was 97.6%.

Collectively, previous studies have demonstrated that although SNP genotyping based on whole-genome resequencing possesses relatively high accuracy, a certain proportion of genotyping errors still exist, especially in the detection of heterozygous loci. In our dataset, we observed 212 discordant calls at heterozygous positions and 128 at homozygous positions. The causes of inconsistent SNP calls between KASP and resequencing could not be clearly identified in this study. Three markers (Chr12_17378039, Chr9_508439 and Chr5_3714662) showed a discordance rate exceeding 10%. Notably, the test materials contained 14 known triploid pear accessions. Short-read resequencing variant calling is challenging for triploids. Duplicated and highly similar genomic regions may produce inaccurate SNP calls and constitute a major potential source of genotype discordance with KASP.

The moderate sequencing depth of the resequencing data (10.72×) may also contribute to the discordance, as heterozygous loci are particularly sensitive to allele read-count fluctuations at this depth. Meanwhile, two independent replicate experiments further confirmed the reliability and stability of these KASP markers. While these three markers were excluded from our final fingerprint set due to high discordance, they remain technically qualified and may be applicable for other purposes.

The PIC values of the 25 KASP markers screened across 269 pear accessions ranged from 0.336 to 0.375, with an average of 0.370. Relevant studies showed that the PIC values of KASP markers were 0.350–0.375 in tea plant (only 43.3% of loci showed high polymorphism) [34], 0.14–0.38 (mean: 0.33) in spring faba bean [35], 0.276–0.500 (mean: 0.375) in *Robinia pseudoacacia* [36], and 0.325–0.375 (mean: 0.350) in fiber-type hemp [37]. Taken together, the polymorphism level of the KASP markers developed in the present study was comparable to that of KASP markers reported in other woody and herbaceous crops, confirming that these markers can be utilized for subsequent genetic diversity evaluation and germplasm discrimination of pear resources.

MAF thresholds for SNP screening in crop germplasm identification vary substantially, with no universal standard available. High-polymorphism SNPs with MAF > 0.4 were selected to maximize discriminatory capacity within this broad germplasm panel. The marker panel developed here is designed specifically for the identification of repository accessions, while adjusted MAF filtering criteria will be needed for materials from narrow breeding backgrounds in future independent studies.

The UPGMA dendrogram revealed relatively low bootstrap support for certain nodes, indicating limited phylogenetic resolution of the 25 SNP markers. Consistent with this, the STRUCTURE analysis should be regarded only as an exploratory assessment of broad genetic relationships rather than a robust inference of population divergence. Furthermore, the 14 triploid accessions were treated as diploids in Ho, PIC and STRUCTURE analyses, as KASP markers cannot distinguish AAB and ABB allelic dosages. This simplification may bias allele-frequency estimates and affect population structure results. Collectively, these population genetic results should be interpreted with caution and regarded as exploratory. Notably, the 12 groups identified as bud mutations or synonymous accessions clustered with high bootstrap support, further confirming their highly similar genetic backgrounds. For instance, ‘Qiuzi’/’Tianqiuzi’ and ‘Nanguo’/’Dananguo’ showed no genotypic differences, as expected given that ‘Tianqiuzi’ is a bud sport of ‘Qiuzi’, and ‘Dananguo’ is a bud sport of ‘Nanguo’. The remaining 10 groups were strongly suspected to be synonymous germplasms (same germplasm, different names), which was further supported by their high phenotypic similarity in the field.

The number of KASP markers directly influences the cost of variety identification, and highly polymorphic sites possess greater discriminatory power. In our study, a set of 14 markers was found to be sufficient for discriminating among 257 pear germplasm accessions. Therefore, we recommend it for routine germplasm screening, while the 22-marker panel can be retained to supply supplementary genotypic information for reference. In the independent panel of 76 pear accessions, nine accessions formed four indistinguishable groups even when all the 22 markers were included. These groups may represent redundantly conserved germplasm in the germplasm repository. Nevertheless, this observation also reveals the practical resolution limit of the marker panel, which should be considered when applying this marker set for pear cultivar identification.

### 3.2. Comparison of KASP and SSR Markers

SSRs and SNPs are two commonly used markers for the identification of DNA fingerprints. SSR markers excel in discriminating samples due to their high degree of polymorphism, which results in a large number of multi-locus genotypes [38]. In contrast, KASP technology can detect up to three genotypes, including two homozygous and one heterozygous genotype [20]. SNP loci exhibit lower allelic diversity because of their bi-allelic nature. The PIC value of SNPs is limited to a range of 0 to 0.5. In our study, the 25 core SNP-KASP markers exhibited relatively high polymorphism, with PIC values between 0.336 and 0.375 and Ho values ranging from 0.33 to 0.56. However, the PIC and Ho values reported for SSR markers in pears are notably higher [39]. This difference in genetic diversity assessment can be attributed to the fact that SSR markers are capable of detecting a greater number of alleles compared to SNPs [40]. Chen et al. [41] found that the resolving power for 150 Chinese jujube accessions’ genetic diversity was similar for 25 SSRs and 4680 SNPs.

SSR markers are a useful tool for genotype identification and genetic diversity evaluation due to their high degree of polymorphism, co-dominance and suitability for automation [42]. Nevertheless, SSR genotyping still suffers from several limitations, such as ambiguous estimation of allele fragment sizes, limited cross-platform compatibility, and suboptimal cost and throughput performance [43]. The KASP assay is easier to genotype, compare and integrate than SSR markers. While SSR markers require electrophoresis steps after PCR, KASP uses direct fluorescence detection on standard qPCR platforms. This streamlined workflow is particularly advantageous in laboratories with routine high-throughput genotyping needs.

Several germplasm resource management agencies have developed both SSR and SNP markers to manage germplasm resources more effectively [44,45,46]. In addition, the combined use of these two markers is necessary to increase the accuracy of DNA fingerprinting and to facilitate communication and collaboration among researchers.

## 4. Materials and Methods

### 4.1. Plant Materials

We selected 269 pear accessions conserved in the China National Germplasm Repository of Pear and Apple (Xingcheng, Liaoning Province, China). These accessions cover all five major cultivated pear species, including 93 *P. bretschneideri*, 66 *P. pyrifolia*, 53 cultivated and wild *P. ussuriensis*, 11 *P. sinkiangensis*, 12 *P. communis*, and 34 hybrid cultivars (Table S6). They originate from the major pear-producing regions of Northeast, North, Northwest and Southwest China and also contain European pear and wild pear resources, exhibiting rich genetic, geographical, and phenotypic diversity.

An independent set of 76 pear accessions (Table S5), which were not included in the 495 resequenced germplasm collection, was used to verify the reliability and discriminatory capacity of the KASP markers.

Genomic DNA was extracted from young leaves using a Plant Genomic DNA Kit (Tiangen, Beijing, China). DNA quality was assessed by 1% agarose gel electrophoresis, and the samples were diluted to 40 ng/µL by a DS-11 ultra-micro spectrophotometer (DeNovix, Wilmington, DE, USA) for KASP genotyping.

### 4.2. SNP Selection

BCFtools v1.10.2 [47] and VCFtools v0.1.16 [48] were used to filter the pear resequencing SNP dataset and obtain SNP statistics. SNPs were screened according to the following criteria: (a) loci are dimorphic; (b) no other mutations occurring within 100 bp upstream or downstream of the polymorphic sites; (c) minor allele frequency (MAF) > 0.4; (d) average sequencing depth ≥ 10; (e) sample missing value = 0; and (f) the flanking sequence of each site was confirmed as single-copy specific by aligning resequencing data to the *P. betuleafolia* reference genome [49] using TBtools-II (Toolbox for Biologists) v2.148 [50]. The optimal loci combination was tested using a Perl script [26]. This set of SNPs enabled complete discrimination of the 495 resequenced pear accessions, as at least one differential SNP genotype could be identified between any two accessions, and the pear dataset used for running scripts is provided in Table S7.

### 4.3. KASP Primer Design and Synthesis

A KASP assay consisted of two forward competitive primers (that recognize different alleles) and one common reverse primer. The primers were designed with the following parameters: (1) GC content < 60%; (2) annealing temperature (Tm) between 55 and 62 °C; and (3) PCR amplification product < 120 bp. The primers were synthesized by Sangon Biotech Co., Ltd. (Shanghai, China). The standard GAAGGTGACCAAGTTCATGCT and GAAGGTCGGAGTCAACGGATT tails were added to the FAM- and HEX-specific allele primers, respectively.

### 4.4. PCR Amplification and Genotyping

KASP reaction system: The KASP primer system consisted of 12 µL (100 µM) of each specific primer, 30 µL of the universal primer (100 µM) and 46 µL of ddH_2_O. PCR was conducted in a 96-well plate, with each well containing 10.14 µL of the reaction mixture composed of 5 µL of DNA at a concentration of 40 ng/µL, 5 µL of 2× KASP Master Mix (Cat. No. KBS-1050-102; LGC, Biosearch Technologies, Hoddesdon, UK) and 0.14 µL of the primer mixture.

The amplification and fluorescent end-point reading were performed using a Bio-Rad CFX96 Touch qPCR System (Bio-Rad, Hercules, CA, USA). Amplifications were carried out under the following conditions: 94 °C for 15 min, followed by 10 cycles of 94 °C for 20 s and 61 °C for 60 s, with the annealing temperature reduced by 0.6 °C per cycle, and then 26 cycles of 94 °C for 20 s and 55 °C for 60 s. SNP markers with a low-GC% content used a slightly different amplification profile: 94 °C for 15 min, 36 cycles of 94 °C for 20 s and 57 °C for 60 s, and a read step at 37 °C for 60 s, following the manufacturer’s recommendation for low-GC content assays [51]. The annealing type and annealing temperature of each marker is shown in Table S2.

If the fluorescence signal was low and scattered, additional cycles were added: 3 cycles of 94 °C for 20 s and 57 °C for 60 s. The allele discrimination mode of Bio-Rad CFX Manager 3.1 software was used for genotype calling. The X-axis and Y-axis corresponded to the signal values of the fluorophores FAM and HEX, respectively. Based on the fluorescence signal values, the samples were clustered and grouped, and the sample clusters were analyzed to ascertain their genotypes.

### 4.5. KASP Result Comparison and Verification

The genotyping results using KASP markers were compared with the corresponding resequencing data from 269 pear germplasm accessions. Loci with inconsistent genotyping results between KASP and resequencing were screened and validated using two independent replicate KASP assays with identical reaction systems and PCR thermal cycling conditions.

### 4.6. Data Analysis

The genotyping data were transformed into coding data: the four bases A, C, G, and T were represented by the numbers 1, 2, 3, and 4, respectively, and missing data were labeled as 9. MAF (minor allele frequency) and PIC (polymorphism information content) were calculated using Excel; PIC was calculated using the formula:

$$PIC=1-\sum_{i=1}^{n}p_{i}^{2}-2\sum_{i<j}p_{i}^{2}p_{j}^{2}$$

where *n* is the number of alleles at the locus, and *p_i_* and *p_j_* represent the frequencies of the *i*-th and *j*-th alleles, respectively.

Observed heterozygosity (Ho) was calculated using GenAlEx 6.501 [52]. The population genetic structure was evaluated using STRUCTURE 2.3.4 [53]. We chose the admixture model with correlated allele frequencies. K values ranging from 1 to 10 were employed to infer the number of clusters in 10 replicate runs. Each run included a burn-in period of 100,000 iterations, followed by 100,000 Markov chain Monte Carlo (MCMC) simulations. The structure results were analyzed using StructureSelector [54] (https://lmme.ac.cn/StructureSelector/index.html, accessed on 20 May 2026) and visualized using the barplot function in R. Genotypes were converted to FASTA format for UPGMA dendrogram construction in MEGA 10.2.6 [55]. Homozygous sites were coded as single base pairs (e.g., GG, CC), heterozygous genotypes were represented by two combined nucleotides (e.g., GC for G/C heterozygotes), and missing data were coded as NN. The tree was built under the maximum composite likelihood model with 1000 bootstrap replicates and visualized using iTOL (https://itol.embl.de/itol.cgi, accessed on 30 June 2026). The screening procedure for core KASP markers is described in the Results Section.

## 5. Conclusions

This study identified and developed 25 KASP markers with abundant polymorphic information and clear genotyping results. After removing three high-discordance loci, the remaining 22 markers were further applied to generate fingerprints of 269 pear accessions, which were subsequently grouped into two clusters based on their geographic origin. Furthermore, we detected ten groups of potential synonym accessions and observed that two sets of bud mutations shared identical genotypes. These findings suggest that SNP-KASP markers are suitable for preliminary screening of pear genetic resources and provide a useful reference for the collection, conservation and rational utilization of pear germplasm.

## 6. Patents

The authors declare that a Chinese invention patent has been granted based on the work reported in this manuscript.

Patent Title: Method and Application for Constructing DNA Fingerprinting of Pear Varieties Based on KASP Marker Combinations.

Patent Number: ZL 2025 1 0041325.2.

Status: Granted.

## Figures and Tables

**Table 1:** Chromosomes, positions, variation types and polymorphism levels of 25 core KASP markers.

No.	Chromosome	Position	SNP REF/ALT	Ho	PIC	MAF	Missing Rate
1	1	16868304	C/T	0.51	0.368	0.41	0
2	2	3483609	G/C	0.46	0.373	0.45	0
3	2	26191031	A/G	0.52	0.374	0.46	0.0074
4	3	4991610	G/C	0.47	0.374	0.46	0
5	4	17841568	A/G	0.48	0.374	0.47	0
6	4	23947857	T/C	0.42	0.362	0.4	0
7	5	3714662	T/C	0.40	0.374	0.47	0.0074
8	6	12064959	C/T	0.40	0.374	0.46	0.0074
9	6	18415632	A/T	0.37	0.362	0.39	0
10	7	5417364	G/T	0.44	0.373	0.44	0
11	8	6167915	G/A	0.37	0.336	0.31	0.0074
12	8	22160552	T/A	0.55	0.373	0.46	0.0074
13	9	508439	A/T	0.49	0.365	0.41	0
14	9	8663266	C/T	0.56	0.375	0.47	0
15	11	5690624	C/T	0.46	0.375	0.5	0.0074
16	11	16140350	T/C	0.43	0.374	0.46	0
17	11	33330733	C/T	0.48	0.373	0.45	0
18	12	3512965	C/A	0.37	0.370	0.4	0
19	12	17378039	C/G	0.35	0.370	0.41	0.0074
20	13	21953151	A/C	0.46	0.375	0.47	0
21	13	26179272	T/C	0.50	0.375	0.48	0
22	14	2476344	A/G	0.42	0.372	0.44	0
23	15	38652743	A/G	0.33	0.372	0.44	0
24	17	1017338	A/G	0.44	0.374	0.47	0.0074
25	17	8230144	T/G	0.5	0.370	0.44	0

## Data Availability

Resequencing genotypes for 25 loci, KASP genotyping data, genotypes of the 76-accession independent validation panel, and the marker-selection dataset are provided in the Supplementary Materials. Raw fluorescence signals from KASP assays were visualized as locus coordinates, and numerical fluorescence values were not exported. Further inquiries can be directed to the corresponding author.

## Supplementary Materials

The following supporting information can be downloaded at https://www.mdpi.com/article/10.3390/plants15203111/s1. Table S1: Chromosomal positions for three loci combinations, 34 synthesized loci, 9 assays failed loci and a set of 14 markers sufficient to identify the 257 pear germplasms; Table S2: SNP information, KASP primer sequences and PCR annealing conditions; Table S3: The resequencing results at 25 loci across 269 pear accessions; Table S4: Q matrix of 269 pear accessions from STRUCTURE analysis (K = 2); Table S5: An independent set of 76 pear accessions and genotype of 22 markers; Table S6: Information for 269 pear accessions: sample code, name, ploidy, species and KASP fingerprint genotype; Table S7: Pear_dataset for running scripts.
